# Supplementary material for: The causal relationship between the human gut microbiota and pyogenic arthritis: a Mendelian randomization study
Source: Front Cell Infect Microbiol. 2024 Nov 26;14:1452480. doi: 10.3389/fcimb.2024.1452480 (PMC11629706; doi:10.3389/fcimb.2024.1452480)
Supplement: Supplementary file 1 [file DataSheet1.zip › mendelian test/ebi-a-GCST90027495.csv_forest.pdf]

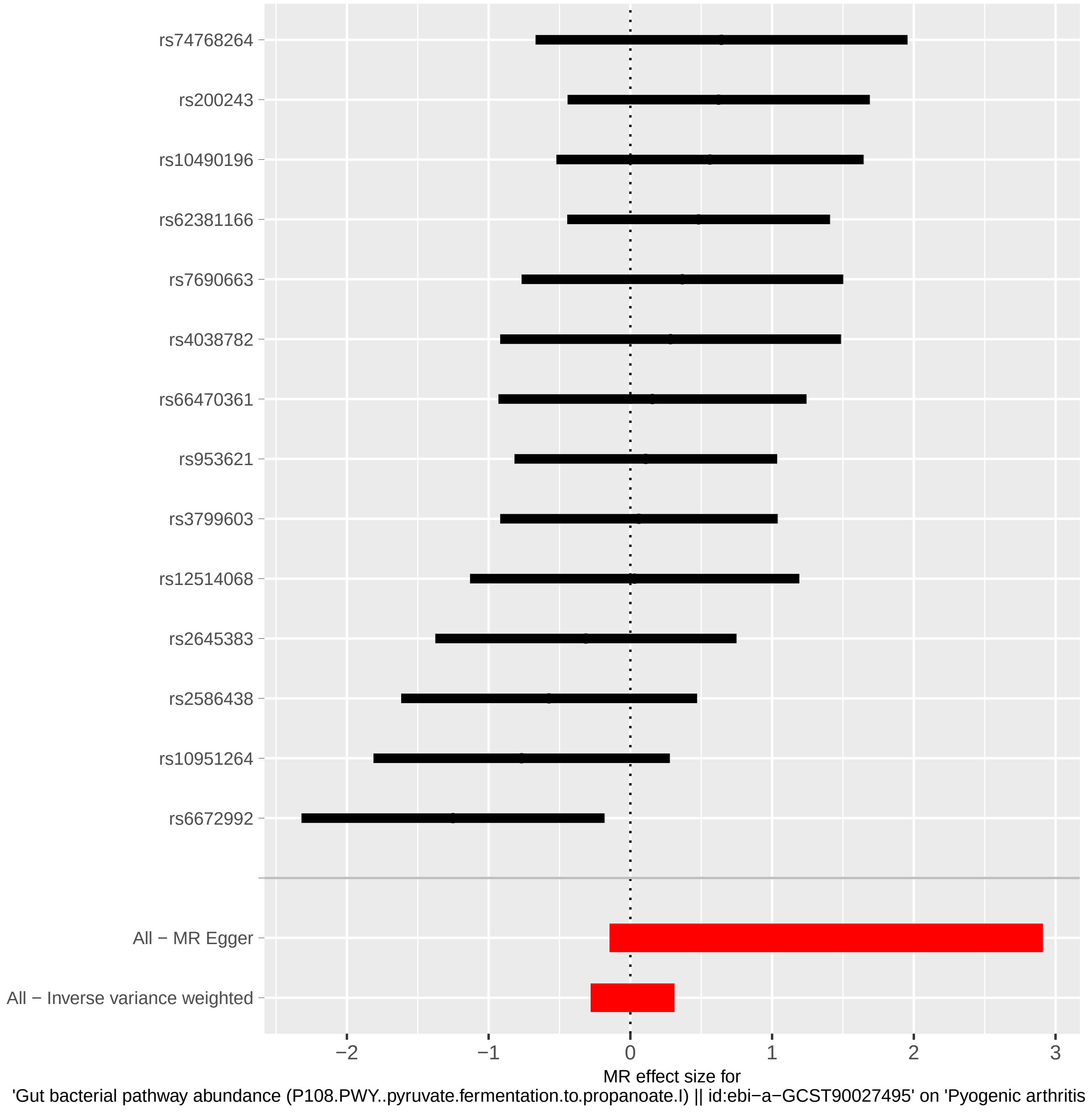
rs74768264
rs200243
rs10490196
rs62381166
rs7690663
rs4038782
rs66470361
rs953621
rs3799603
rs12514068
rs2645383
rs2586438
rs10951264
rs6672992
All − MR Egger
All − Inverse variance weighted
−2
−1
0
1
2
3
MR effect size for
'Gut bacterial pathway abundance (P108.PWY..pyruvate.fermentation.to.propanoate.I) || id:ebi−a−GCST90027495' on 'Pyogenic arthritis
